# Social Isolation and Suicidal Risk Among Elderly Psychiatric Outpatients

**DOI:** 10.1192/j.eurpsy.2026.11589

**Published:** 2026-07-31

**Authors:** N. Smaoui, M. Barkallah, S. Omri, I. Gassara, R. Feki, N. Charfi, M. Maalej, L. Zouari

**Affiliations:** Psychiatry Department “C”, Hedi Chaker Hospital, Sfax, Tunisia

## Abstract

**Introduction:**

Social isolation and feelings of loneliness are recognized as major contributors to suicidal behavior among the elderly.

**Objectives:**

We aimed to explore the relationship between perceived loneliness and suicidal risk in older adults followed in outpatient psychiatry.

**Methods:**

A cross-sectional study was conducted among patients aged 65 years and older attending psychiatric consultations. Data collection included self-reported measures of loneliness, the 3-point Loneliness Scale and social isolation assessed through the Social Isolation Scale, which comprises a Social Link Index (ILS) quantifying social connections and a Close Relationship Index (IRE) evaluating the qualitative dimension of interpersonal bonds. Suicidal history and characteristics were explored in detail and complemented by the Ducher Suicide Risk Scale.

**Results:**

Seventy-three patients were included (male/female ratio = 1.08). A high prevalence of loneliness was observed, with 72% of participants reporting feeling lonely “often” or “sometimes.” More than half (51%) stated they had felt lonely during most of the past week, and 58% reported intensified loneliness during specific periods of the year, particularly in the evening (44%) and during summer holidays (23%).

The median score on the 3-point Loneliness Scale was 5 (IQR = 3–7). Regarding social isolation, 33% of participants were classified as socially isolated according to the ILS, while only 34% reported having close relationships (IRE).

According to the Ducher Suicide Risk Scale, 40% of patients expressed no death-related thoughts, whereas the remaining 60% showed varying degrees of suicidal ideation, from transient thoughts to active intent.

A significantly higher suicidal risk was correlated with greater subjective loneliness (p < 0.0001), higher scores on the 3-point Loneliness Scale (p < 0.0001), increased social isolation (p < 0.0001), and the absence of close relationships (p < 0.0001).

**Conclusions:**

Loneliness was highly prevalent among elderly psychiatric outpatients and appeared closely intertwined with suicidal risk. The predominance of impulsive and reactive suicidal behaviors underscores the need for systematic screening of social isolation and emotional distress in this vulnerable population.

**Disclosure of Interest:**

None Declared

